# Weaning from extracorporeal membrane oxygenation: experience with dexmedetomidine in seven adult ARDS patients

**DOI:** 10.1186/cc14565

**Published:** 2015-03-16

**Authors:** M Cozzolino, A Franci, A Peris, L Tadini Buoninsegni, B Loriga

**Affiliations:** 1 A.O.U. Careggi, Firenze, Italy

## Introduction

Sedation in the ICU is a basic therapeutic procedure to increase tolerance of invasive treatments and reduce discomfort. Extracorporeal membrane oxygenation (ECMO) is a highly invasive treatment and prolonged sedation may be required. Patients undergoing ECMO represent a challenge with respect to sedation. Initially, deep sedation may be required to optimize ventilation and circuit-patient flows and to minimize oxygen consumption. The other critical phase is represented by weaning from ECMO support. Optimal sedation is not clearly defined, moreover there are no data on sedation practices with dexmedetomidine (DEX) in adult patients undergoing ECMO. In contrast to other sedatives, DEX has analgosedative effects without respiratory depression, and could be useful to facilitate spontaneous respiratory activity during recovery from sedation.

## Methods

We investigate the role of DEX as a sedative agent used during recovery from deep sedation and weaning from extracorporeal support in patients on vv-ECMO. From May 2014 to October 2014 we prospectively enrolled seven patients affected by ARDS of different etiologies treated with vv-ECMO. The mean age was 53.7 ± 7.9 years and the mean ICU stay was 21.4 ± 11.5 days. Initially, all patients were sedated with association of opioids and GABA receptor agonists, following the internal protocol. At the time of weaning from ECMO, ruled out cardiovascular instability, we started the administration of DEX (0.7 μg/kg/hour, without initial bolus) with progressive decrease of the dose of other sedative drugs.

## Results

The mean duration of DEX infusion was 6.1 ± 4.8 days. Except for one patient, who received DEX as a single drug after suspension of other sedatives, a low-dose infusion of another sedative (<50% compared with initial dose) was maintained. Three patients presented adverse events: two bradycardia and one hypotension. In four patients DEX was discontinued after recovery of respiratory function; in two patients deeper sedation for ventilatory dyssynchrony was needed so other sedative drugs were started. Only in one patient was the drug suspended for extreme bradycardia, resolved after suspension.

## Conclusion

In our study, DEX allowed the reduction of doses of other sedative drugs during weaning from vv-ECMO; this may lead to a cooperative sedation, promoting spontaneous breathing. Side effects described and the cost-benefit ratio must still be verified extensively in patients during weaning from ECMO.

